# Whole Cigarette Smoke Increased the Expression of TLRs, HBDs, and Proinflammory Cytokines by Human Gingival Epithelial Cells through Different Signaling Pathways

**DOI:** 10.1371/journal.pone.0052614

**Published:** 2012-12-28

**Authors:** Abdelhabib Semlali, Chmielewski Witoled, Mohammed Alanazi, Mahmoud Rouabhia

**Affiliations:** 1 Groupe de Recherche en Écologie Buccale, Département de stomatologie, Faculté de Médecine Dentaire, Université Laval, Québec, Québec, Canada; 2 Genome Research Chair, Department of Biochemistry, College of Science King Saud University, Riyadh, Kingdom of Saudi Arabia; National Jewish Health, United States of America

## Abstract

The gingival epithelium is becoming known as a regulator of the oral innate immune responses to a variety of insults such as bacteria and chemicals, including those chemicals found in cigarette smoke. We investigated the effects of whole cigarette smoke on cell-surface-expressed Toll-like receptors (TLR)-2, −4 and −6, human β-defensin (HBD) and proinflammatory cytokine expression and production in primary human gingival epithelial cells. Whole cigarette smoke was shown to increase TLR2, TLR4 and TLR6 expression. Cigarette smoke led to ERK1/2, p38 and JNK phosphorylation in conjunction with nuclear factor-κB (NFκB) translocation into the nucleus. TLR expression following cigarette smoke exposure was down regulated by the use of ERK1/2, p38, JNK MAP kinases, and NFκB inhibitors, suggesting the involvement of these signaling pathways in the cellular response against cigarette smoke. Cigarette smoke also promoted HBD2, HBD3, IL-1β, and IL-6 expression through the ERK1/2 and NFκB pathways. Interestingly, the modulation of TLR, HBD, and cytokine expression was maintained long after the gingival epithelial cells were exposed to smoke. By promoting TLR, HBDs, and proinflammatory cytokine expression and production, cigarette smoke may contribute to innate immunity dysregulation, which may have a negative effect on human health.

## Introduction

Tobacco smoking has been associated with an increased incidence of bacterial infections, chronic pulmonary obstructive disease (COPD), asthma and bronchitis [1]–[3]. Smoking is also a major risk factor in the development of various cancers, including oral cancers that affect a significant number of people worldwide each year [4].

The frequency of oral cancer is often indicative of the patterns of use of tobacco products [5], [6]. A dose-response relationship has been established between the amount of tobacco product used and oral cancer development [7]–[9]. Indeed, smoking severely impairs several functions of both the alveolar macrophages and airway epithelial cells, including the inhibition of lipolysaccharide (LPS)-induced expression of TNF-α, IL-1β, and IL-6 [10]–[13], microbicidal activity, and phagocytosis [14]. These observations suggest that smoking suppresses the ability of the host to develop the innate immune response to infection in the oral cavity [15], [16].

Oral mucosa is under constant alert due to physical insults, invading microbes, and chemicals. Epithelial cells, key actors of innate immunity, not only play an important role in maintaining the physical barrier between the host and the environment but also actively participate in tissue innate immunity [17], [18] by specifically expressing certain receptors, including toll-like receptors (TLRs) that are involved in host immune response [19], [20].

TLRs can be expressed on the cell surface (TLR1–6) or expressed in intracellular vesicles such as endosomes and the endoplasmic reticulum (TLR3, TLR7–9). These TLRs are involved in the recognition of multiple foreign agents, including microbes and toxic substances [21], [22]. TLRs also contain a cytoplasmic tail domain that is homologous to the interleukin-1 receptor and is responsible for initiating various intracellular signaling cascades. These signaling cascades include the activation of NFκB, a crucial transcription factor that promotes the expression of such immune response genes as cytokines, chemokines, and co-stimulatory and adhesion molecules [21], [22].

Several TLRs have been identified in humans, and each one recognizes a specific PAMP. Epithelial cells, notably, have been shown to express various TLRs, including TLRs 1–6 and 9 [23]. These TLRs are used by the epithelial cells to sense multiple negative and positive stimuli [24]. For example, TLR2 expression in keratinocytes by *S. aureus* or its components, peptidoglycan and lipoteichoic acid, resulted in the activation of NFκB and subsequent production of the neutrophil chemotactic factors IL-8 and iNOS [25]. Previous studies have demonstrated that TLR3 activation by its ligand dsRNA (poly I:C) in human keratinocytes induces the production of IL-8, TNFα, IL-18, and type I interferon (IFNα/β) as well as the development of Th1-type immune responses [26]. Furthermore, TLR5 activation in human keratinocytes by its ligand, flagellin, resulted in the production of TNFα, IL-8, and the antimicrobial peptides human β-defensin 2 and 3 (HBD2 and HBD3) [27], [28]. Lebre *et al.* showed that TLR3 and TLR9 activation in keratinocytes led to the selective production of the chemokines CXCL9 and CXCL10, which promoted memory T cell and type I interferon responses [29]. Epithelial cells also sensed *C. albicans* infection through TLR activation and the production of the antimicrobial peptides HBD2 and HBD3 [30].

TLR activation is dependent on the common TLR adaptor protein MyD88 [31], [32]. When recruited, this protein leads to the phosphorylation of downstream kinases such as IL-1R-associated kinase (IRAK)-1 and IRAK4, and TGF-β-associated kinase (TAK)1, as well as p38, JNK, and ERK MAP Kinases (MAPKs) [33]. MAPKs mediate several cell functions, including the phosphorylation of the transcription factors NFκB and AP-1 and the transcription of proinflammatory and chemotactic cytokines [34]–[36].

A dysregulation of TLR functions may lead to a decrease of innate immunity and possible cancer development. This response dysregulation may occur through cellular exposure to foreign agents such as tobacco smoke. Indeed, it has been shown that cigarette smoke condensate induces structural and functional changes in the bronchial epithelium by altering cell signaling pathways [37], [38]. In addition to genetic insults, cigarette smoke constituents have also been shown to activate biochemical pathways that are associated with apoptosis, cell cycle progression, and cell growth [39].

Based on the data generated with bronchial epithelium, we hypothesized that cigarette smoke may also have a direct effect on the innate immune response of the gingival epithelium by affecting TLR expression and the production of proinflammatory cytokines and antimicrobial peptides, such as HBDs. The primary goal of this study was thus to investigate whether whole cigarette smoke affected the expression of cell-surface-expressed TLRs (2, 4 and 6), proinflammatory cytokines, and HBD-2 and -3 by normal human gingival epithelial cells. Our second focus was to examine the downstream signaling pathways (ERK1/2, p38, JNK, and NFκB ) in these cells following the exposure to whole cigarette smoke.

## Materials and Methods

### General Reagents

1R3F cigarettes were purchased from the Kentucky Tobacco Research & Development Center (Orlando, FL, USA). ELISA kits were obtained from R&D Systems (Minneapolis, MN, USA). The Beta defensin staining kit was obtained from Phoenix Pharmaceuticals Inc. (Burlingame, CA, USA). Primers were obtained from Medicorp Inc. (Montréal, QC, Canada). PD98059, an inhibitor of ERK1/2 kinase, and SB202190, a specific inhibitor for p38 kinase, were obtained from EMD Millipore (Billerica, MA, USA), while SP600125, a JNK inhibitor, and IKK-2 inhibitor to block NFκB were obtained from Enzo Life Sciences Inc. (Farmingdale, NY, USA). Anti-ERK1/2 (Clone 631122) (1∶1000), anti-phosphorylated ERK1/ERK2 (AF 108) (1∶1000), and anti-p38 (9212) (1∶1000) were purchased R&D Systems; anti-phosphorylated p38 (Thr180/Tyr182, 92115) (1∶1000) was purchased from Cell signaling (Cell Signaling Technology, Inc. Danvers, MA, USA); and anti-JNK1/2 (sc 1648) (1∶1000), anti-phosphorylated JNK1/2 (sc 6254) (1∶1000) and anti-NFκB (anti-p65, sc 109) (1∶50) were from Santa Cruz Biotechnology (Santa Cruz, CA, USA). Anti–β-actin (1∶10 000) and peroxidase-conjugated antibodies (1∶1000) were from Sigma-Aldrich Canada Ltd. (Oakville, ON, Canada), while Alexa-Fluor-488-conjugated secondary antibodies were from Life Technologies Inc. (Burlington, ON, Canada).

### Culture of Human Gingival Epithelial Cells

Normal human gingival epithelial cells (ScienCell Research Laboratories; Carlsbad, CA, USA). were cultured in Dulbecco’s modified Eagle’s–Ham’s F12 (3∶1; DMEH) medium supplemented with 5 µg/mL of human transferrin, 2×10^−9^ m of 3,3′,5′-triiodo-l-thyronine, 0.4 µg/mL of hydrocortisone, 10 ng/mL of epidermal growth factor, 100 IU/mL of penicillin G, and 10% fetal bovine serum. The medium was changed three times a week. When the culture reached 90% confluence, the cells were detached from the flasks with a 0.05% trypsin–0.1% ethylenediaminetetraacetic acid (EDTA) solution, were washed twice and were resuspended in DMEH-supplemented medium at a final concentration of 10^6^ cells/mL. Cells at the third passage were used to perform the experiments.

### Whole Cigarette Smoke Cell Challenge Assays

The gingival epithelial cells were seeded onto 35 mm diameter×10 mm deep Petri dishes at 3×10^5^ cells per plate in DMEH medium and were incubated in a humidified atmosphere containing 5% CO_2_ at 37°C. When the culture reached approximately 80% confluence, the epithelial cell cultures were overlaid with a thin layer of culture medium (1.5 ml) and were placed inside a smoke chamber [40]. Briefly, a cigarette was placed into one end of a tube linked to the chamber. On the other end, a second tube linked the chamber to a standard vacuum. This setup allowed the cigarette smoke to penetrate inside the chamber, where the quantity/volume of smoke was controlled by a specific valve. The cultures were placed into the chamber under sterile conditions, and the chamber was hermetically covered prior to burning the cigarette. Cultures were exposed to the smoke of one whole cigarette for 15 or 30 min, after which time the cells were fed fresh medium and were cultured for various durations; control cells were not exposed to smoke. The temperature inside the chamber remained stable. Following each culture period, the epithelial cell cultures underwent analysis.

### Quantitative Real-time RT-PCR

Total cellular RNA was extracted with the Illustra RNAspin Mini (GE Health Care UK Ltd., Buckingham, UK). The concentration, purity, and quality of the isolated RNA were all determined using the Experian system and RNA StdSens analysis kit according to the manufacturer’s instructions (Bio-Rad, Hercules, CA, USA).

RNA (1 µg of each sample) was reverse transcribed into cDNA using Maloney murine leukemia virus (M-MLV) reverse transcriptase (Invitrogen Life Technologies, Mississauga, ON, Canada) and random hexamers (Amersham Pharmacia Biotech, Inc., Baie d’Urfé, QC, Canada). The conditions for the preparation of the cDNA templates for PCR analysis were 10 min at 65°C, 1 h at 37°C, and 10 min at 65°C. Quantitative PCR (qPCR) was carried out as previously described [41]. The quantity of mRNA transcripts was measured using the Bio-Rad CFX96 real-time PCR detection system. Reactions were performed using a PCR supermix from Bio-Rad (iQ SYBR Green supermix). Primers (Table 1) were added to the reaction mix at a final concentration of 250 nM. Five microliters of each cDNA sample was added to a 20 µl PCR mixture containing 12.5 µl of iQ SYBR Green supermix (Bio-Rad), 0.5 µl of specific primers (TLR2, TLR4, TLR6, HBD2, HBD3, IL-1β, IL-6, or GAPDH) (Medicorp, Inc., Montréal, QC, Canada), and 7 µl of RNase/DNase-free water (MP Biomedicals, Solon, OH, USA). Each reaction was performed in a Bio-Rad MyCycler Thermal Cycler. For the qPCR, the CT was automatically determined using the accompanying Bio-Rad CFX manager. The thermocycling conditions for the TLRs were established as 5 min at 95°C, followed by 35 cycles of 15 s at 95°C, 30 s at 60°C, and 30 s at 72°C, with each reaction performed in triplicate. For the HBDs and interleukins, the thermocycling conditions were 3 min at 95°C, followed by 30 cycles of 10 s at 95°C, 10 s at 63°C, and 30 s at 72°C, with each reaction also performed in triplicate. The specificity of each primer pair was verified by the presence of a single melting temperature peak. GAPDH produced uniform expression levels varying by less than 0.5 CTs between sample conditions and was therefore used as a reference gene for this study. The amplified products were run on an agarose gel to confirm that no spurious products were amplified during the cycles. The results were analyzed using the 2^−ΔΔCt^ (Livak) relative expression method.

**Table 1:** Primer sequences used for the qRT-PCR.

Gene name	Primers sequences	Product size (bp)
**TLR-2**	sense : 5′-GCCTCTCCAAGGAAGAATCC-3′antisense : 5′-TCCTGTTGTTGGACAGGTCA-3′	144
**TLR-4**	sense : 5′-AATCTAGAGCACTTGGACCTTTCC-3′antisense : 5′-GGGTTCAGGGACAGGTCTAAAGA-3′	116
**TLR-6**	sense : 5′-CATCCTATTGTGAGTTTCAGGCAT-3′antisense : 5′-GCTTCATAGCACTACATCCCAAG-3′	121
**β2-defensin**	sense : 5′-TGTGGTCTCCCTGGAACAAAAT-3′antisense : 5′-GTCGCACGTCTCTGATGAGG-3′	105
**β3-defensin**	sense : 5′-CTTCTGTTTGCTTTGCTCTTCCT-3′antisense : 5′-CTGTTCCTCCTTTGGAAGGCA-3′	138
**IL-1β**	sense : 5′-CTGTCCTGCGTGTTGAAAGA-3′antisense : 5′-TTGGGTAATTTTTGGGATCTACA-3′	69
**IL-6**	sense : 5′-TCTCCACAAGCGCCTTCG-3′antisense : 5′-CTCAGGGCTGAGATGCCG-3′	203
**GAPDH**	sense : 5′-GGTATCGTCGAAGGACTCATGAC-3′antisense : 5′-ATGCCAGTGAGCTTCCCGTTCAGC-3′	180

### FACS Analysis

Gingival epithelial cells (3 × 10^5^) were cultured in DMEH medium as described above. When the culture reached approximately 80% confluence, the experimental cell cultures were challenged with whole cigarette smoke for 15 min. The culture medium was refreshed, and the cells were incubated for an additional 24 h in a 5% CO_2_ humid atmosphere at 37°C. To determine TLR expression, epithelial cells were detached from the culture plates by trypsinization for 2 min. The cell suspensions were then washed twice with DMEM culture medium and were used to determine TLR2, TLR4, or TLR6 surface expression. To do so, cells (10^6^) were incubated for 1 h with anti-TLR2 (C19, sc 8690), anti-TLR4 (C18, sc8694), or anti-TLR6 (N18, sc 5657) Goat-IgG polyclonal antibodies at a final concentration of 10 µg/ml (Santa Cruz Biotechnology, Santa Cruz, CA, USA). The cells were then washed twice with sterile PBS/2% bovine serum albumin (BSA) and were incubated with FITC-conjugated mouse anti-goat IgG antibody in the dark for 45 min on ice, after which the cells were washed thrice with PBS/2% BSA, resuspended in 500 µl of PBS, and analyzed with an EPICS ELITE ESP ﬂow cytometer (Beckman-Coulter, Miami, FL).

### Cytokine and β-defensin Quantification

Experimental gingival epithelial cells at 80% confluence were exposed to whole cigarette smoke for either 15 or 30 min, after which the medium was refreshed and cells were cultured for 24 h. The supernatant from each condition was collected to determine the IL-1β, IL-6, HBD2, and HBD3 levels. The supernatants were collected in tubes containing 1 µl of a protease inhibitor cocktail (Sigma-Aldrich) and immediately filtered through 0.22 µm filters and used to measure the mediator levels by sandwich enzyme-linked immunosorbent assays (ELISA). ELISA plates were read at 450 nm and were analyzed using a Microplate Reader Model 680 (Bio-Rad, USA). The minimum detectable concentrations were under 1 pg/ml for IL-1β, 0.7 pg/ml for IL-6, 0.7 pg/ml for HBD2, and 1.5 pg/ml for HBD3, as reported by the manufacturer. Each experiment was repeated four times and the means+SD were calculated and presented.

### Western Blot Analysis of Signaling Pathways

Gingival epithelial cells at 80% confluence were cultured in serum-free medium for 24 h prior to exposure to whole cigarette smoke for 15 or 30 min. Following each exposure period, the medium was refreshed, and the cells were cultured for various time points. The cells were then detached with trypsin, centrifuged and resuspended in lysis buffer containing 25 mM of Tris-HCl, pH 8.0, 150 mM of NaCl, 1 mM of EDTA, 10% glycerol, 0.1% SDS, 0.05% sodium deoxycholate, and 1% Triton X-100. Added to the homogenized samples were 0.5 mM of proteinase inhibitor PMSF and the phosphatase inhibitors NaF (10 mM) and Na_3_VO_4_ (2 mM). Following 1 h of incubation at 4°C, the samples were centrifuged at >12,000 g for 2 min, after which the supernatants were collected and subsequently stored at −20°C.

The protein concentration was determined using the Bradford assay. Equal amounts of total protein (20 µg) in reducing sample buffer (61.5 mM of Tris, 2% SDS, 10% glycerol, and 100 mM of DTT) were boiled for 5 min and migrated using a 4% stacking gel, followed by 7%, 10%, or 12% acrylamide SDS-PAGE. The gels were then transferred to PVDF membranes with Tris-glycine refrigerated transfer buffer (25 mM of Tris, 19.2 mM of glycine, 20% methanol, and 100 mM of Na_3_VO_4_) for 1 h at 100 V. The blots were then incubated overnight with the appropriate primary antibody to total and phosphorylated ERK1/2, total and phosphorylated JNK, or total and phosphorylated p38, after which the membranes were washed and incubated for 1 h in the appropriate peroxidase-conjugated secondary antibody. Antibody detection was performed using the ECL detection system (Amersham Pharmacia Biotech, Piscataway, NJ, USA) according to the manufacturer’s instructions. Luminescence was visualized by autoradiography.

### NFκB Cell Labeling

Human oral epithelial cells (5×10^5^) were grown in chamber slides for 24 h and then exposed to cigarette smoke for 15 min. Following exposure, the cells were cultured for 24 h and subsequently fixed in 60% acetone/40% methanol at −20°C for 30 min, after which the cells were permeabilized with 0.2% Triton X-100 in PBS at room temperature for 10 min prior to antibody staining. The cultures were then overlaid with NFκB antibody anti-p65 (sc 109) for 1 h. Following extensive washing, the cells were overlaid with FITC-conjugated secondary antibody for 45 min. The slides were then washed and the nuclei were stained with Hoechst dye (1 µg/ml) for 15 min at room temperature. The slides were examined by two independent pesrons. At lest ten randomized fields were observed under a fluorescence microscope and photographed. Representative photos were presented.

### Inhibition of MAP Kinases and NFκB Signaling Pathways

To assess the contribution of MAP kinases in the cigarette smoke-mediated innate immunity mediators, 80% confluent cell cultures were pre-incubated for 45 min with 10 µM (non-toxic for the cells) of commercially available inhibitors to p38 (SB202190), ERK (PD98059), IKK-2, and JNK (SP600125) prior to exposure to whole cigarette smoke for 15 and 30 min. The cells were then post-cultured for 6 h, after which the total cellular RNA was extracted and used to perform qRT-PCR for different genes, as described above.

### Gingival Epithelial Cell Passage (SubCultures)

To investigate the effect of cigarette smoke on gingival epithelial cell subcultures, we exposed cells (80% confluence) to cigarette smoke for 15 min, refreshed the medium, and cultured for 24 h, after which the cells were detached from the culture plates using trypsin, counted, seeded at 10^6^ cells per flask, and subcultured up to 50% confluence (4 days). These cells were then detached and subcultured for 3 days. This step was repeated one more time, resulting in a total subculture period of 10 days. Following the third subculture, the cells were used to extract the total RNA, as described previously. The extracted RNA from each condition was subsequently used to assess TLR2, HBD2, and IL-6 gene expression.

### Statistical Analyses

Each experiment was performed at least four times, with experimental values expressed as the means ± SD. The statistical significance of the differences between the control (non-exposed) and the experimental (cigarette smoke exposed) values was determined with a one-way ANOVA. *Posteriori* comparisons were performed using Tukey’s method. Normality and variance assumptions were verified using the Shapiro-Wilk test and the Brown and Forsythe test, respectively. All of the assumptions were fulfilled. *P* values were declared significant at ≤0.05. Data were analyzed using the SAS version 8.2 statistical package (SAS Institute Inc., Cary, NC, USA).

## Results

### Whole Cigarette Smoke Increased TLR2, TLR4, and TLR6 Expression by the Gingival Epithelial Cells

**Figure 1:**
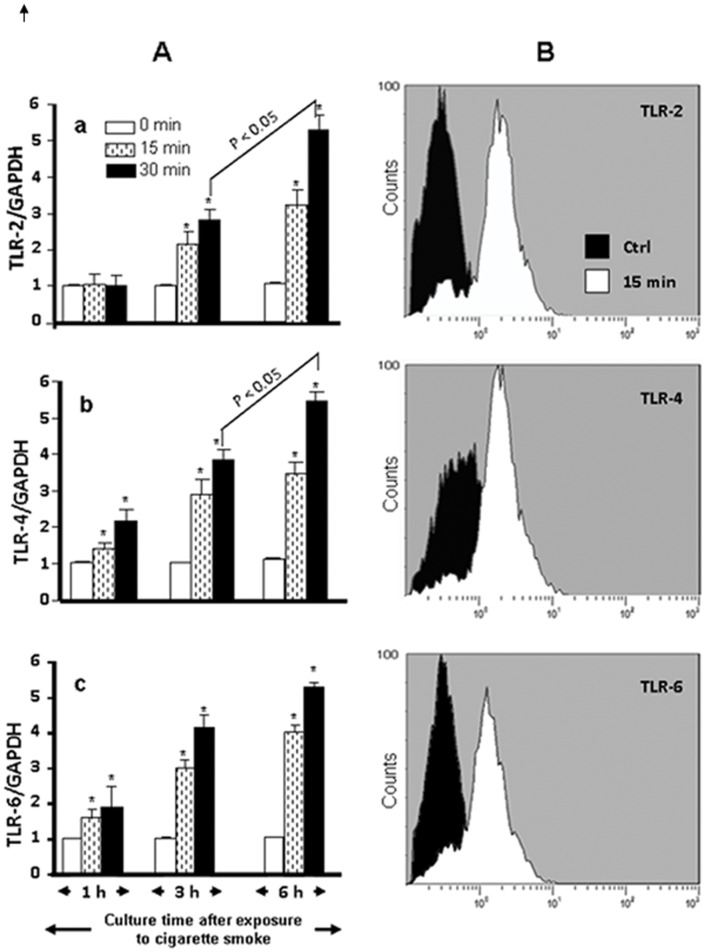
Whole cigarette smoke induced Toll-like receptor TLR2, 4, and 6 expression in normal human gingival epithelial cells. Gingival cells were exposed to whole cigarette smoke for 15 or 30 min or left untreated and subsequently cultured for various time periods prior to TLR gene expression analysis by qRT-PCR. TLR expression level at the cell membrane level was evaluated by flow cytometry. Representative histogram plot of each TLR expression: dark curve = non-exposed control cells; white curve = smoke-exposed cells. (n = 6); (Panel A), gene expression levels; (Panel B), protein expression. *, p<0.001.

As shown in Figure 1A, the exposure of normal human gingival epithelial cells to whole cigarette smoke for 15 or 30 min followed by a culture for 1, 3, or 6 h led to an increase of mRNA expression. TLR2 expression levels significantly (p<0.05) increased following exposure for 15 and 30 min compared to those observed in the non-stimulated cells. It is interesting to note that following the 30-min exposure to cigarette smoke, the 6-hour culture showed greater (p<0.001) TLR2 expression than did the 3-hour culture, suggesting that the effect of the cigarette smoke on the epithelial cells was maintained.

TLR2 was not the only Toll receptor affected by cigarette smoke, as TLR4 expression was also up regulated. This increase was detectable 1 h post-exposure. Cell exposure for 15 or 30 min showed a significant (p<0.05) increase of TLR4 mRNA expression compared to that observed in the controls (cells not exposed to the smoke). This TLR4 mRNA expression was not linked to the exposure time. TLR6 showed the same pattern of expression as TLR4. A 1-hour culture after cell exposure to smoke recorded approximately two times more TLR6 expression than with the non-exposed cells. Greater TLR6 mRNA expression levels were obtained at 3 and 6 h post-exposure. Thus, the data show that whole cigarette smoke increased TLR2, TLR4, and TLR6 mRNA expression.

To confirm these data, we studied the TLR protein expression using flow cytometry. As shown in Figure 1B, following exposure to cigarette smoke for 15 min and culture for 24 h, elevated TLR2, TLR4, and TLR6 protein expression compared to non-exposed control cells was recorded.

### Cigarette Smoke Exposure Induced Human β-defensin 2 and 3 Expression and Production by Normal Human Gingival Epithelial Cells

**Figure 2:**
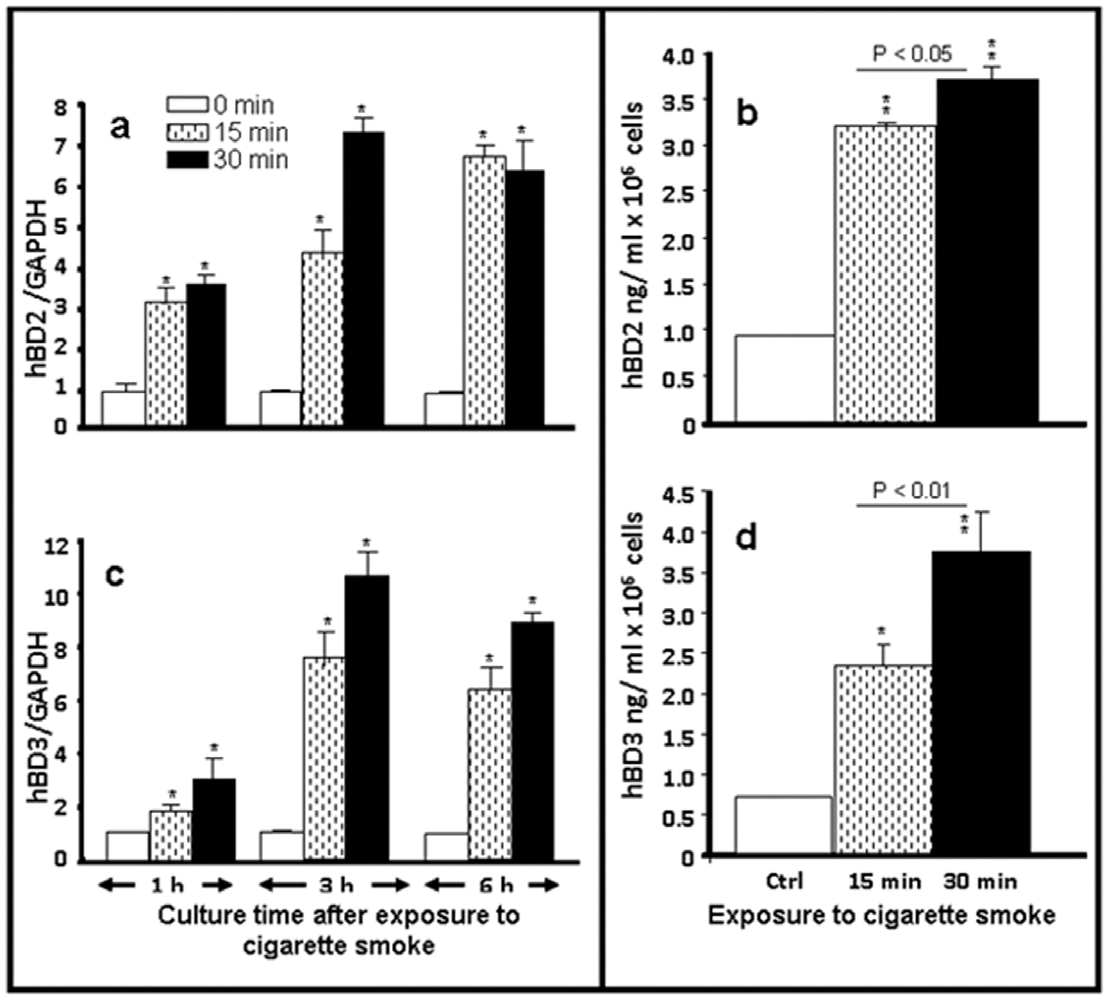
Human β-defensin-2 and -3 gene and protein expression levels increased when normal human gingival epithelial cells were exposed to cigarette smoke. Confluent (80%) gingival epithelial cell cultures were exposed to whole cigarette smoke for 15 or 30 min or left untreated and subsequently cultured for various time periods prior to gene expression analysis by qRT-PCR (3a and 3c). Data are expressed as the means+SD (n = 5); *, p<0.01. The β-defensin secretion levels were quantified by ELISA (3b and 3d) of the supernatant of the cells exposed to cigarette smoke and post-cultured for 24 h. Data are expressed the means+SD, (n = 6). **, p<0.0001.

It is well known that gingival epithelial cells express various TLRs including TLRs 2–6 and 9 [16]. Mammalian cells use these TLRs as sensors to detect various external agents such as microbes and chemicals. This TLR signaling results in innate immune responses that involve the release of various mediators such as β-defensins [29]. Because whole cigarette smoke was shown to increase TLR expression and production by gingival epithelial cells, we investigated the β-defensin (HBD) status. Figure 2a shows that HBD2 mRNA expression significantly (p<0.05) increased following cellular exposure to cigarette smoke for 15 or 30 min. This increase was maintained for 1, 3 and 6 h post-exposure to the smoke. Protein levels confirmed this increased HBD2 mRNA expression. Indeed, ELISA measurement of HBD2 at 24 h post-exposure to cigarette smoke demonstrated a significant (p<0.001) increase of HBD2 levels compared to those recorded in the non-exposed gingival cell cultures (Fig. 2b). Furthermore, the level of HBD2 obtained with 30 min of exposure was significantly (p<0.05) higher than with 15 min of exposure. Thus, whole cigarette smoke increased HBD2 expression and production by the gingival epithelial cells. These results raised questions regarding HBD3 expression and production. The answers are reported in Figure 2c, which shows that exposure for 15 or 30 min to whole cigarette smoke followed by culture for 1, 3 or 6 h led to increased HBD3 mRNA expression. The greatest effect continued to be observed with the 30-min exposure. Furthermore, the 3 h post-exposure time point gave the highest levels of HBD3 mRNA expression. This modulatory effect of the smoke was confirmed by assays of protein production. As shown in Figure 2d, ELISA analysis revealed a significant (p<0.01) increase of HBD3 levels in the culture supernatant of the gingival epithelial cells exposed to whole cigarette smoke compared to the non-exposed cultures. In addition, the levels of HBD3 obtained after 30 min of exposure were significantly (p<0.01) higher than those obtained after 15 min. Overall, both HBD2 and HBD3 expression was up regulated following gingival exposure to cigarette smoke.

### Cigarette Smoke Exposure Induced IL-1β and IL-6 Expression and Secretion by the Normal Human Gingival Epithelial Cells

**Figure 3:**
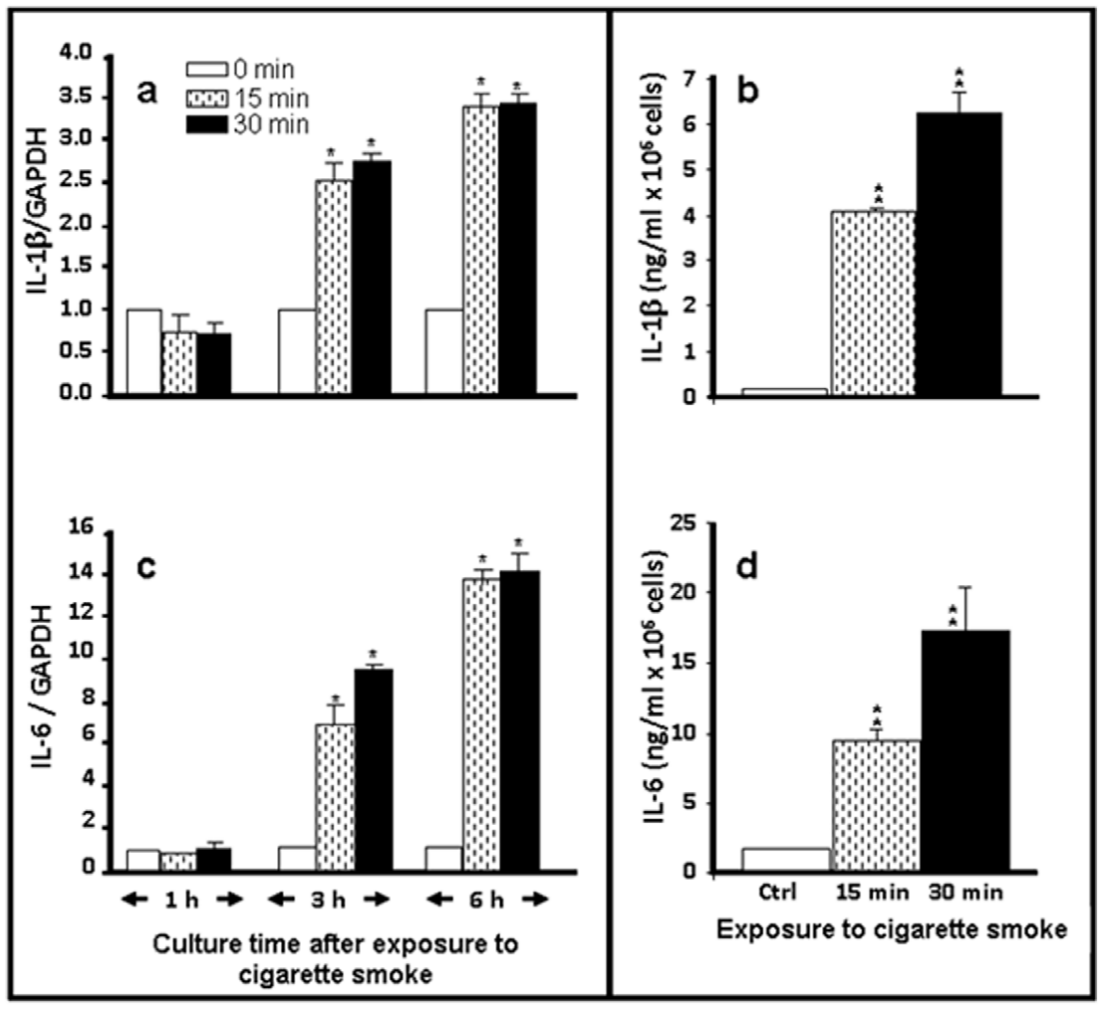
Human IL-1β and IL-6 gene and protein expression levels increased after normal human gingival epithelial cells were exposed to whole cigarette smoke. Confluent (80%) gingival epithelial cell cultures were exposed to whole cigarette smoke for 15 or 30 min or left untreated and then cultured for various time periods prior to gene expression analysis by qRT-PCR (5a and 5c). Data are expressed as the means+SD (n = 5); *, p<0.01. IL-1β and IL-6 levels were quantified by ELISA (5b and 5d) of the cells exposed to cigarette smoke and post-cultured for 24 h. Data are expressed the means+SD, (n = 6). **, p<0.001.

Because the activation of TLRs led to pro-inflammatory cytokine expression [42], and because we demonstrated that cigarette smoke stimulated the expression of these TLRs by gingival epithelial cells, we proceeded to examine IL-1β and IL-6 expression and secretion. As shown in Figure 3a, exposure of the gingival epithelial cells to whole cigarette smoke for 15 or 30 min followed by culture for different time periods led to an increase of IL-1β mRNA expression after 3 and 6 h post-culture. This significant (p<0.01) increase was comparable in both exposure periods. The increased IL-1β mRNA expression was confirmed by protein secretion. Figure 3b shows the significant (p<0.001) increase of IL-1β level at 15 or 30 min of exposure to cigarette smoke and subsequent culture for 24 h. Furthermore, it should be noted that the longer the exposure time to cigarette smoke, the greater were the IL-1β secretion levels by the gingival epithelial cells. Similar observations were made with IL-6. Figure 3c and d show significant (p<0.01) increases of IL-6 mRNA expression and secretion at 3 and 6 h post-exposure in the smoke-exposed cells. The same range of IL-6 increase was obtained at 15 and 30 min of exposure to whole cigarette smoke. Overall, cigarette smoke promoted IL-1β and IL-6 expression by gingival epithelial cells.

### Cigarette Smoke Promoted the Phosphorylation of ERK1/2, p38 and JNK, as well as NFκB Translocation into the Nucleus

**Figure 4:**
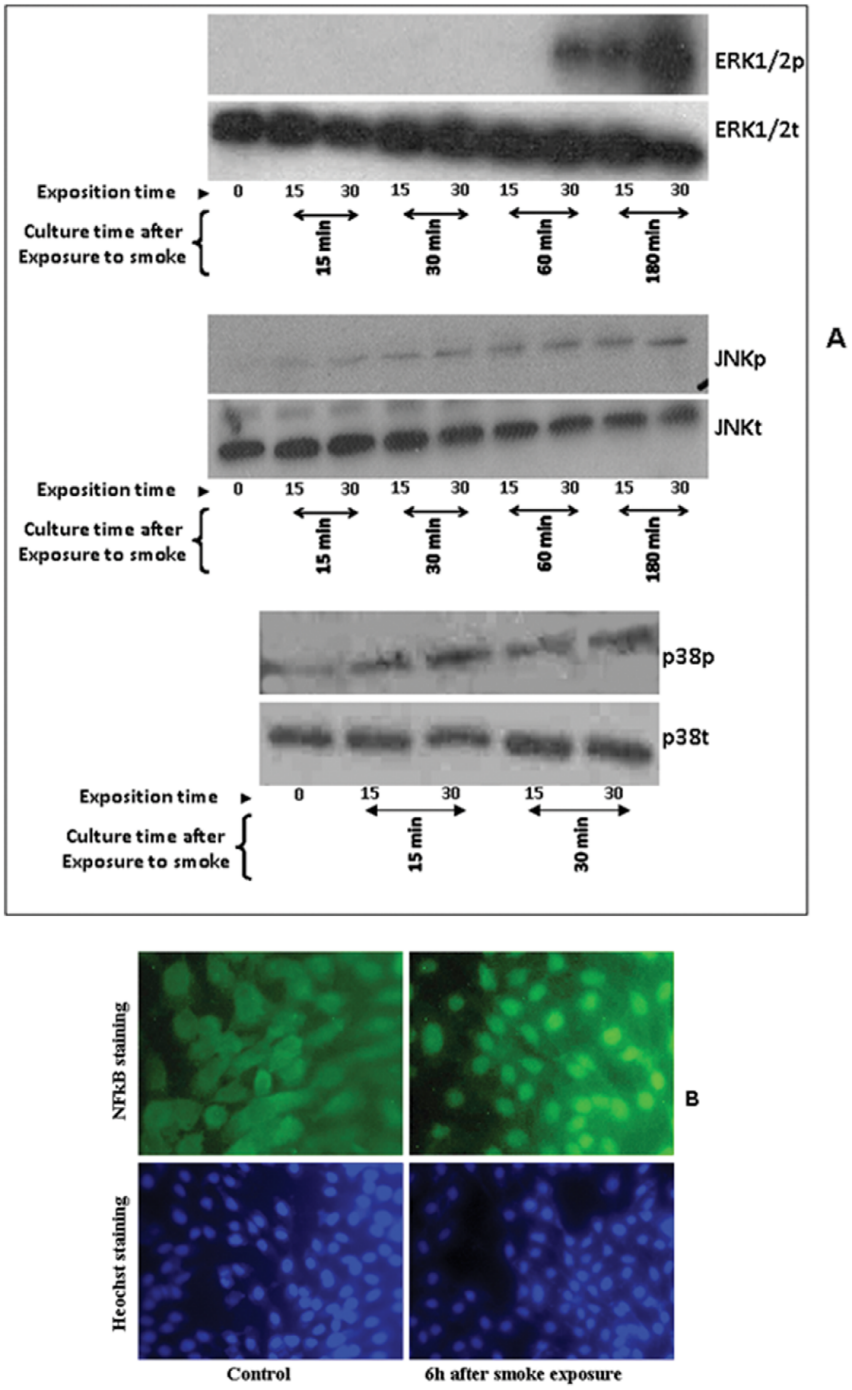
Whole cigarette smoke promoted ERK1/2 and JNK phosphorylation and NFκB translocation from the cytoplasm to the nucleus of gingival epithelial cells. Confluent (80%) cell cultures were exposed to whole cigarette smoke for 15 or 30 min or left untreated; cells were then cultured for various time periods prior to Western blot and immunofluorescence analyses. Panel (A) shows the Western blot results. Equal amounts of whole-cell protein lysates were separated on 12% gels, transferred to a PVDF membrane, and detected using total and phosphor-specific antibodies. The scanned gel shown is representative of four independent experiments. Panel (B) shows the immunofluorescence results. Cells (500 000) were seeded onto glass slides and cultured for 24 h, then exposed to whole cigarette smoke for 15 min. Following exposure, the cells were cultured for 24 h, then permeabilized and stained with primary anti- NFκB monoclonal antibody and FITC secondary antibody. The cell nucleus was revealed by Hoechst (n = 4).

Having shown that cigarette smoke increased TLR, HBD, and proinflammatory cytokine expression and secretion through the MAP kinase pathway that included ERK and JNK, we hypothesized that cigarette smoke would also promote TLR-induced epithelial cell proinflammatory responses by activating downstream signaling intermediates. To address this possibility, we first analyzed the effect of smoking on ERK1/2, p38, and JNK phosphorylation. As shown in Figure 4A, ERK1/2 was phosphorylated beginning at 60 min with a high level at 180 min, JNK was phosphorylated beginning at 30 min with a high level at 180 min, and p38 was phosphorylated beginning at 15 min with high level at 30 min post-exposure to cigarette smoke. This result confirms the TLR modulation by cigarette smoke through the ERK132, JNK and p38 MAP kinase. Figure 4A shows that this phosphorylation was promoted by cigarette smoke as early as 30 min post-exposure to cigarette smoke and remained detectable even after 180 min post-exposure. However, at the 180 min culture time point, JNK phosphorylation was lower than ERK1/2 phosphorylation. For p38, phosphorylation was promoted by cigarette smoke as early as 15 min, with a high level at 30 min post-exposure to cigarette smoke (Fig. 4). This p38 phosphorylation was maintained at 60 and 180 min post exposure to cigarette smoke (data not shown).

Next, we investigated the nuclear translocation of the NFκB transcription factor by immunofluorescence staining of NFκB. As shown in Figure 4B, cigarette smoke stimulated the nuclei-labeled cells with NFκB in the gingival epithelial cell cultures. The percentage of NFκB-positive nuclei in the smoke-exposed culture was as high as 60% compared to that recorded by the non-exposed cells. This result suggests a role of the NFκB pathway following the exposure of the gingival epithelial cell to cigarette smoke.

### Cigarette Smoke Modulates TLRs, HBDs and Proinflammatory Cytokines through Specific Pathways

**Figure 5:**
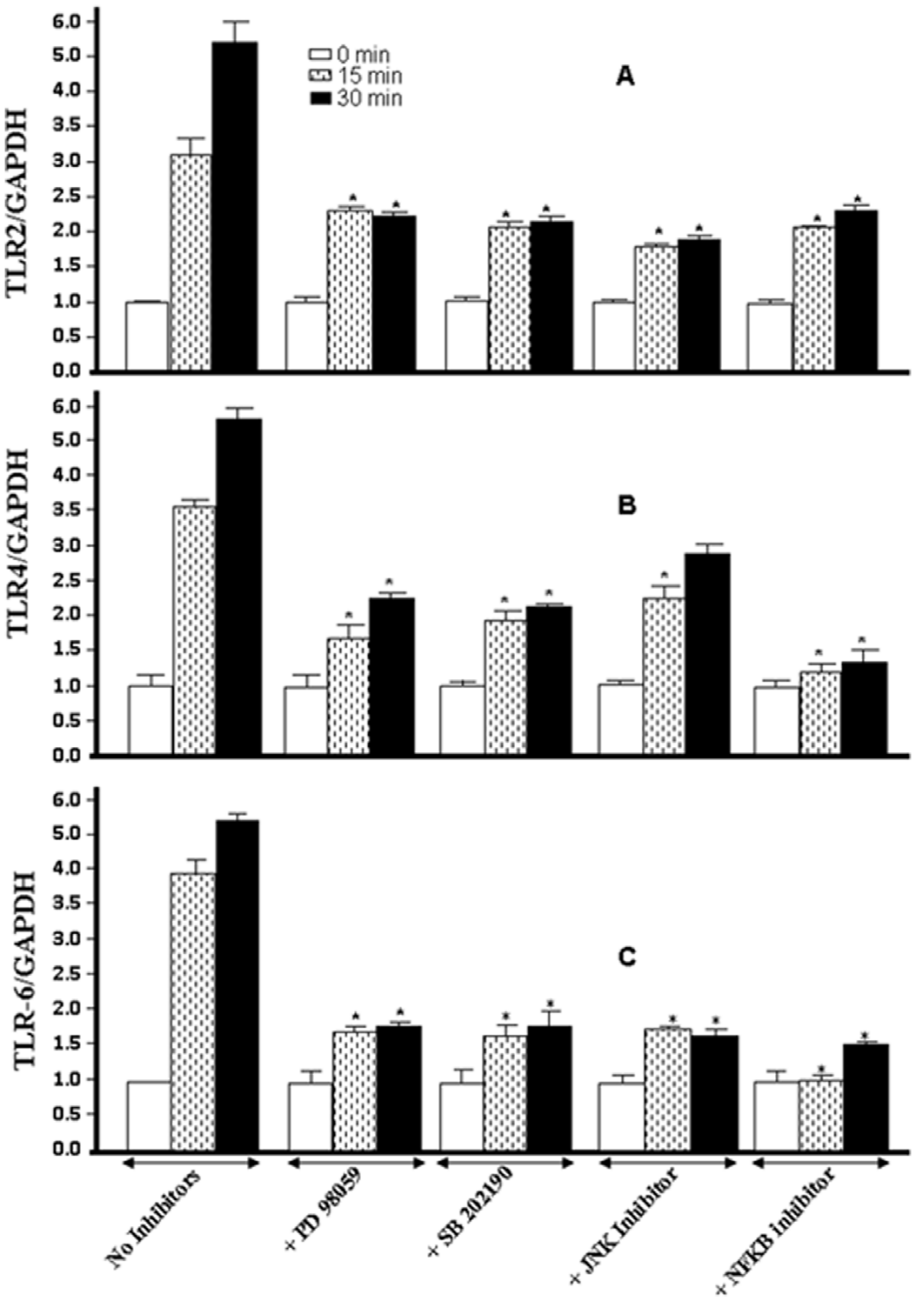
TLR modulation by whole cigarette smoke leads to ERK1/2, p38, JNK, MAP kinase and NFκB activations. Confluent (80%) gingival epithelial cell cultures were incubated with 10 µM of ERK inhibitor (PD98059), 10 µM of p38 inhibitor (SB202190), 10 µM of JNK inhibitor (SP600125), or 10 µM of NFκB inhibitor (IKK-2) for 45 min before exposure to cigarette smoke for 15 or 30 min. The cells were then incubated 6 h, after which the total RNA was extracted, and TLR expression was analyzed by qRT-PCR (n = 6). *, p<0.001 when comparing the values obtained in the presence and absence of inhibitor.

To confirm the signaling pathway activated by cigarette smoke and it effect on TLRs, HBDs and proinflammatory cytokine expression, we used specific signaling pathway inhibitors. As shown in Figure 5A, the addition of an ERK1/2 inhibitor (PD98059) significantly (p<0.01) reduced TLR2 expression compared to that observed in the cells cultured without inhibitor. The same results were obtained when using inhibitors of p38 (SB202190), JNK (SP600125), and NFκB (IKK-2). These results suggest a role of different MAP kinase pathways in TLR2 expression by gingival epithelial cells following exposure to whole cigarette smoke. Analyses of the signaling pathways engaged in TLR4 expression revealed that ERK1/2, p38, JNK MAP kinases, and NFκB were involved, as specific inhibitors to each of these kinases significantly reduced TLR4 mRNA expression (Fig. 5B). However, the NFκB pathway appeared to be the most involved in the smoke-exposed gingival cells. In contrast, the JNK pathway was moderately involved in only those cultures exposed for 15 min to cigarette smoke. Additionally, our results (Fig. 5C) indicate that the same signaling pathways (ERK1/2, p38, JNK, and NFκB) were modulated by the TLR6 increase following gingival epithelial cell exposure to the smoke.

**Figure 6:**
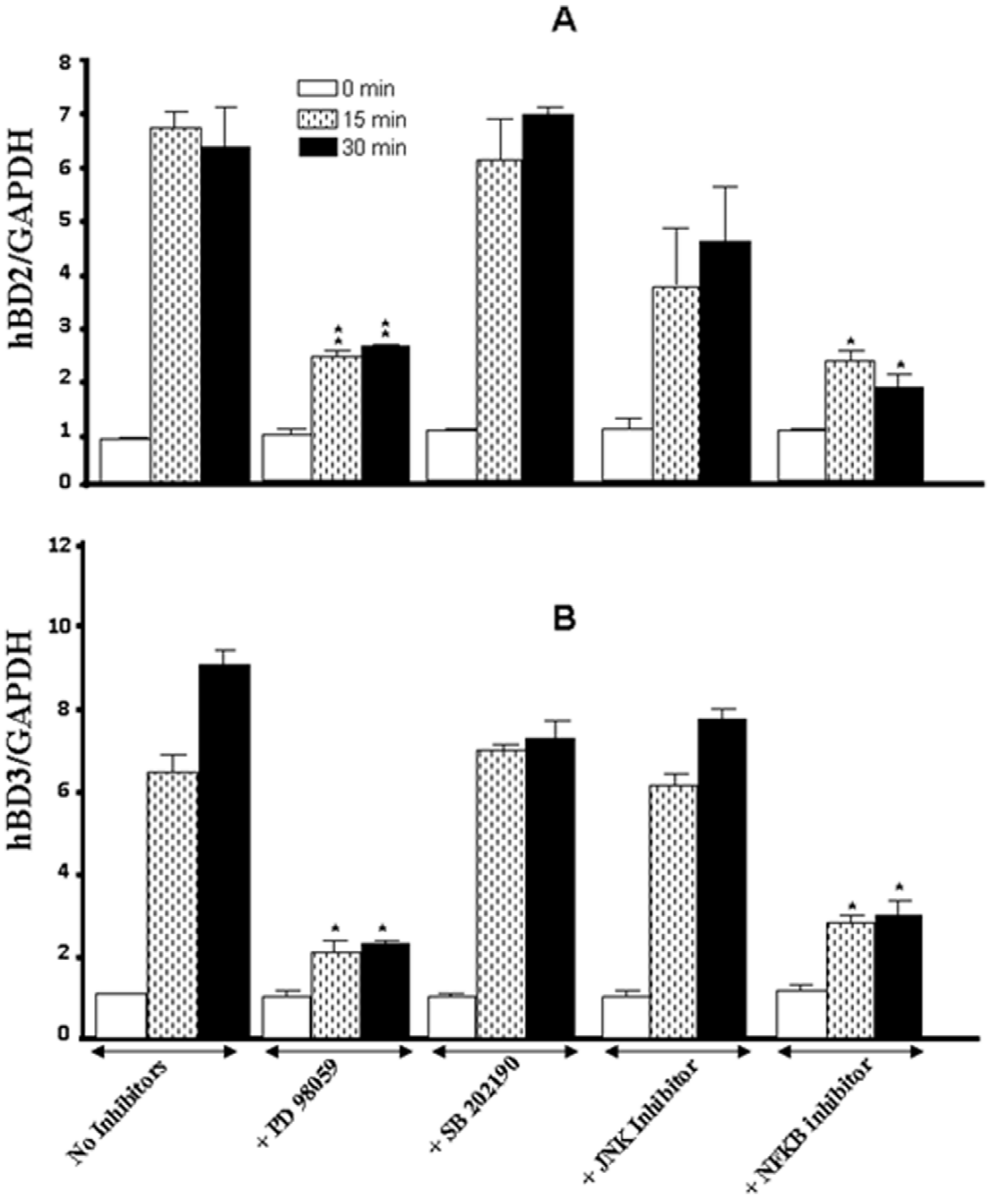
Whole cigarette smoke promoted human β-defensin-2 and -3 expression through the ERK1/2 MAP kinase and NFκB signaling pathways. Confluent (80%) gingival epithelial cell cultures were incubated with 10 µM of ERK inhibitor (PD98059), 10 µM of p38 inhibitor (SB202190), 10 µM of JNK inhibitor (SP600125), or 10 µM of NFκB inhibitor (IKK-2) for 45 min before exposure to cigarette smoke for 15 or 30 min. Six hours later, total RNA was extracted, and HBD gene expression was analyzed by qRT-PCR (n = 6). *, p<0.05; **, p<0.01 when comparing values obtained in the presence and absence of inhibitor. The results are expressed as the means+SD, (n = 5).

**Figure 7:**
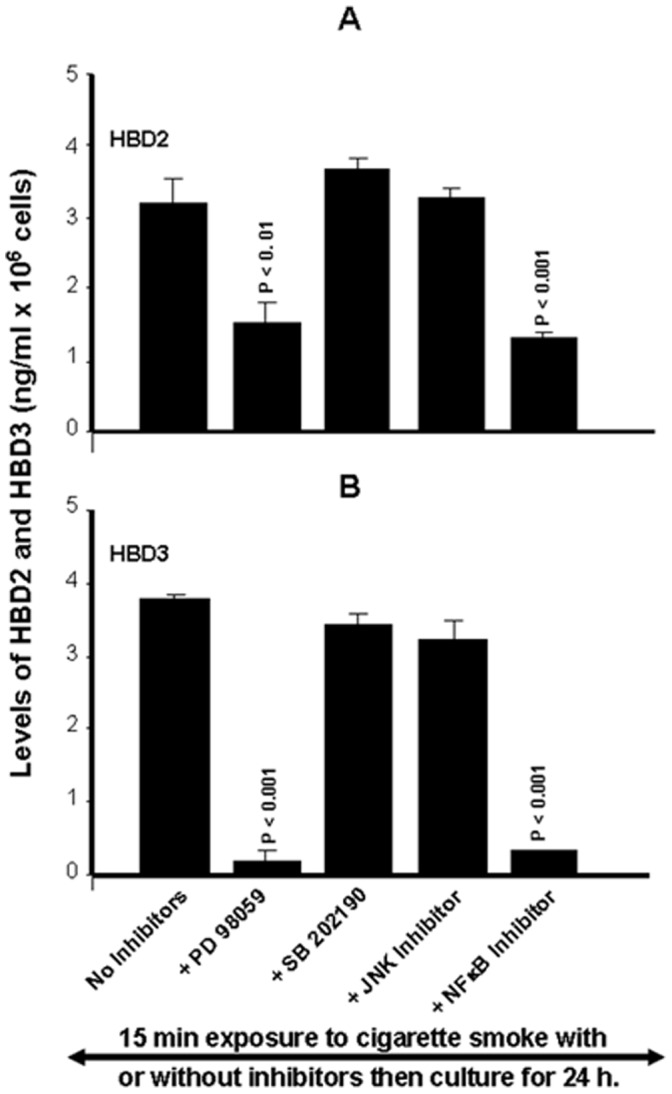
Whole cigarette smoke promoted human β-defensin-2 and -3 secretions through the ERK1/2 MAP kinase and NFκB signaling pathways. Confluent (80%) gingival epithelial cell cultures were incubated with 10 µM of ERK inhibitor (PD98059), 10 µM of p38 inhibitor (SB202190), 10 µM of JNK inhibitor (SP600125), or 10 µM of NF-κB inhibitor (IKK-2) for 45 min before exposure or not to cigarette smoke for 15 mn. 24 hours later, supernatants were collected and used to measure the β-defensin secretion levels by ELISA kits. Data are expressed the means+SD, (n = 4).

Modulation of HBD by cigarette smoke also involved specific signaling pathways. As shown in Figure 6A, only the inhibition of ERK or NFκB had a significant (p<0.01) impact on the effect by the cigarette smoke, as both of these MAP kinase inhibitors had a similar profile of action against the cigarette smoke-induced HBD2 mRNA expression in gingival epithelial cells. The blockage of ERK and NFκB followed by exposure of the cells to cigarette smoke for 30 min produced an effect that was similar to that observed with the 15-min exposure, and a similar observation was made with HBD3. As shown in Figure 6B, only the inhibition of ERK and NF-κB inhibited the cigarette smoke-induced HBD3 mRNA expression. Both ERK and NFκB inhibitors displayed a significant (p<0.01) and similar behavior against cigarette smoke-induced HBD3 expression by gingival epithelial cells. The inhibitory effects at 15 and 30 min of exposure were thus comparable (Fig. 6B). Protein analyses confirmed the specific involvement of ERK or NFκB. As shown in Figure 7A, the inhibition of ERK or NFκB had a significant (p<0.01) impact on the effect by the cigarette smoke, as both of these MAP kinase inhibitors had a similar profile of action against the cigarette smoke-induced HBD2 protein secretion by gingival epithelial cells. The blockage of ERK and NFκB followed by exposure of the cells to cigarette smoke for 15 min produced a decrease of hBD2 secretion (Fig. 7A). The same results were obtained with 30 min exposure to cigarette smoke (data not shown). Similar observations were made with hBD3. Only the use of inhibition of ERK and NFκB inhibitors led to a significant (p<0.01) down regulation of hBD3 secretion by gingival epithelial cells following exposure to cigarette smoke for 15 min (Fig. 7B).

**Figure 8:**
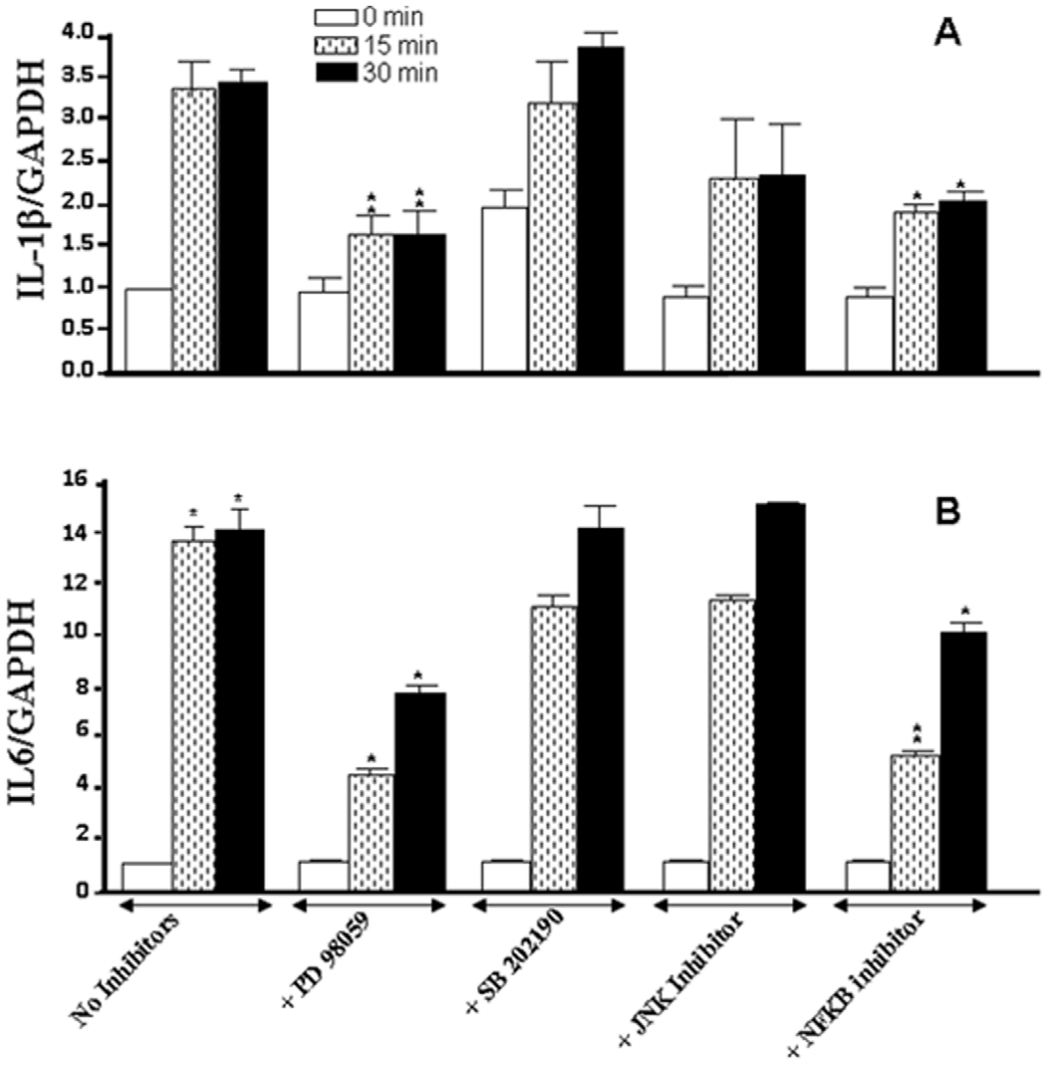
Whole cigarette smoke promoted IL-1β and IL-6 expression through the ERK1/2 and NFκB signaling pathways. Confluent (80%) gingival epithelial cell cultures were incubated with 10 µM of ERK inhibitor (PD98059), 10 µM of p38 inhibitor (SB202190), 10 µM of JNK inhibitor (SP600125), or 10 µM of NFκB inhibitor (IKK-2) for 45 min before exposure to cigarette smoke for 15 or 30 min. Six hours later, total RNA was extracted, and cytokine gene expression was analyzed by qRT-PCR (n = 6). *, p<0.05; **, p<0.01 when comparing values obtained in the presence and absence of inhibitor. The results are expressed as the means+SD, (n = 5).

**Figure 9:**
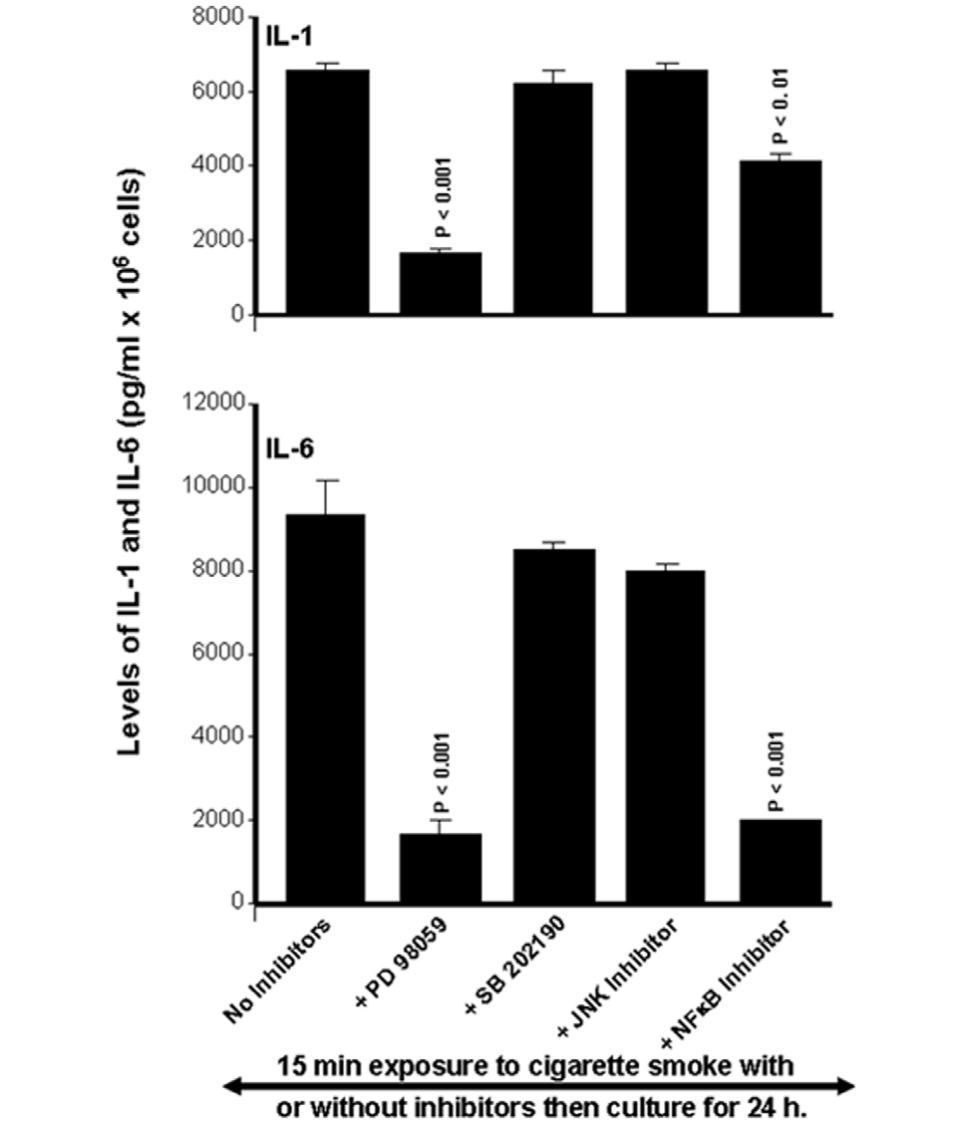
Whole cigarette smoke promoted human IL-1β and IL-6 secretions through the ERK1/2 MAP kinase and NFκB signaling pathways. Confluent (80%) gingival epithelial cell cultures were incubated with 10 µM of ERK inhibitor (PD98059), 10 µM of p38 inhibitor (SB202190), 10 µM of JNK inhibitor (SP600125), or 10 µM of NFκB inhibitor (IKK-2) for 45 min before exposure or not to cigarette smoke for 15 min. supernatants were collected 24 h later and used to measure the secretion of IL-1β and IL-6 by ELISA kits. Data are expressed the means+SD, (n = 4).

Proinflammatory cytokines were also modulated by cigarette smoke through specific signaling pathways. As shown in Figure 8A, the cell cultures with MAP kinase or NFκB inhibitors and exposure to cigarette smoke demonstrated that only the inhibition of ERK or NFκB caused significant (p<0.01) inhibition of IL-1β mRNA expression. These inhibitory effects on the ERK1/2 and NFκB were similar whether the cells were exposed to the smoke for 15 or 30 min. The effect with IL-1β was also similarly observed with IL-6. Figure 8B shows that the inhibition of ERK or NFκB, but not p38 or JNK, caused a decrease of smoke-induced IL-6 mRNA expression. Protein analyses supported the mRNA data. Indeed, the use of specific inhibitors to ERK or NFκB, and exposure for 15 min to cigarette smoke showed significant (p<0.01) inhibition of the secretion of IL-1β and IL-6 by gingival epithelial cells (Fig. 9). Similar results were obtained with ERK or NFκB inhibitors then cell exposure for 30 min to cigarette smoke (data not shown).

### The Effect of Cigarette Smoke on TLR, HBD2, and IL-6 Expression was Maintained Over Gingival Epithelial Cell Passages

**Figure 10:**
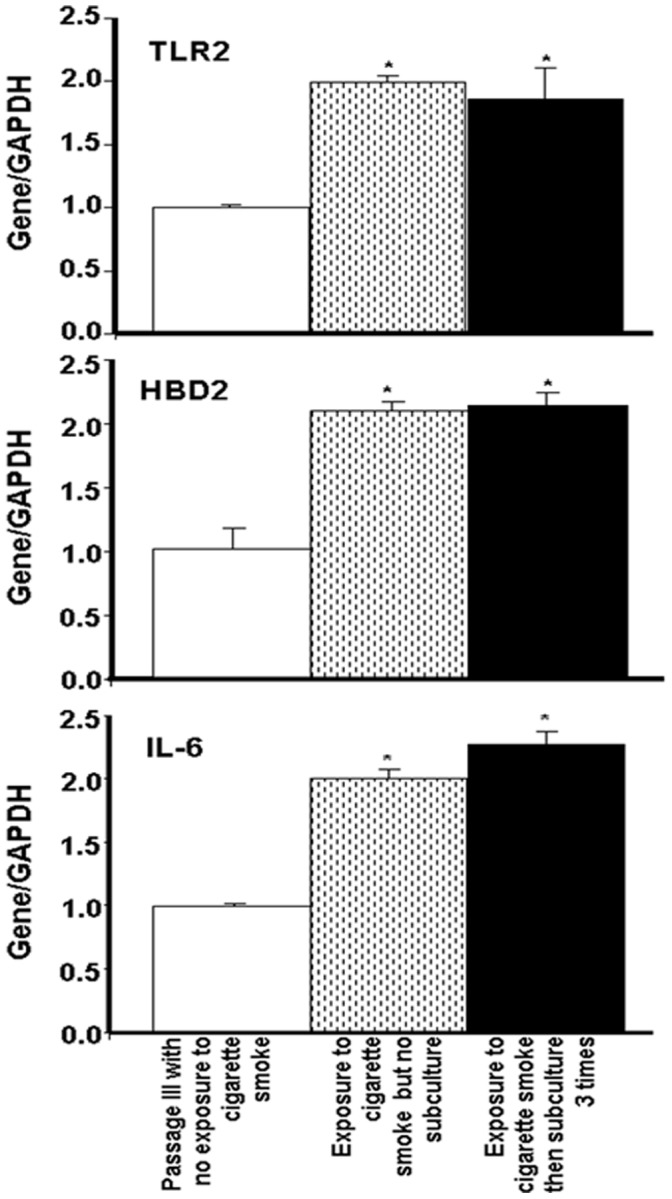
The effect of whole cigarette smoke on TLR2, human β-defensin-2, and IL-6 expression by gingival epithelial cells was maintained over multiple cell passages. Confluent (80%) gingival epithelial cell cultures were exposed to whole cigarette smoke for 15 min or left untreated, followed by culture for 3 h, after which the cells were detached and used either to extract total RNA or to subculture for 4 days. This subculture was repeated twice every 3 days for a total culture period of 10 days. Extracted RNAs from the different conditions were analyzed by qRT-PCR. Data are expressed as the means+SD (n = 5); *, p<0.0001.

**Figure 11:**
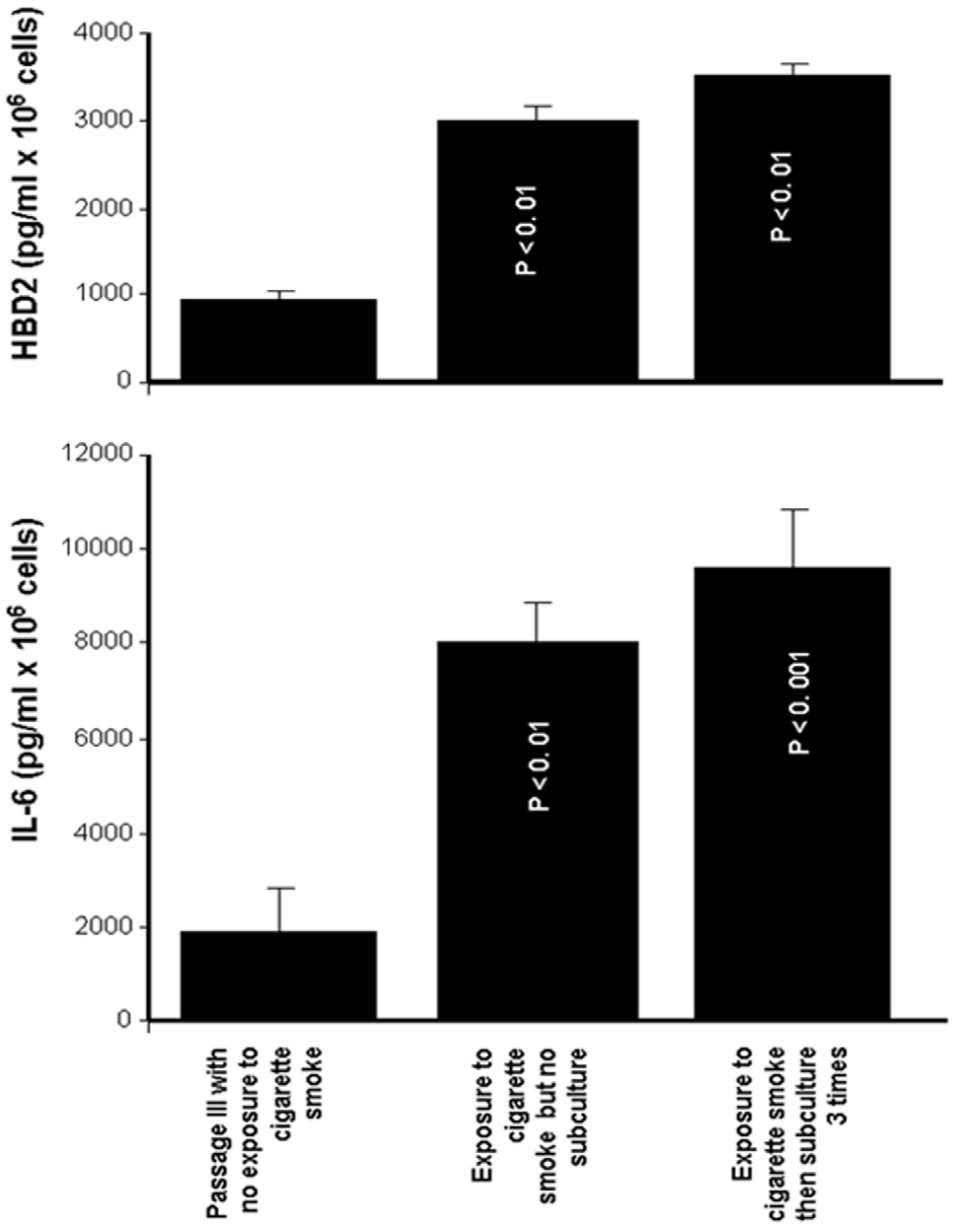
Human β-defensin-2 and IL-6 secretions by gingival epithelial cells were maintained over multiple cell passages following exposure to cigarette smoke. Confluent (80%) gingival epithelial cell cultures were exposed to whole cigarette smoke for 15 min or left untreated, followed by culture for 24 h, after which supernatants were collected and used to determine the levels of HBD2 or IL-6 by ELISA. Data are expressed as the means+SD (n = 5).

As the cigarette smoke increased TLR, HBD, and proinflammatory cytokine expression by the gingival epithelial cells, we hypothesized that the effect of the smoke was transmitted from one cell generation to another. To address this possibility, epithelial cells were exposed to cigarette smoke, cultured for 4 days, and then trypsinized three times for each three-day subculture period to ensure continuous exponential growth. At the end of the third subculturing, total RNA was extracted, and TLR2, HBD2, and IL-6 mRNA expression was analyzed. As shown in Figure 10, subculturing the cells that were exposed to cigarette smoke did not contribute to reducing the level of TLR2 mRNA expression by the gingival epithelial cells. Indeed, even after three passages, the TLR2 mRNA expression remained significantly (p<0.01) high in those cells exposed to cigarette smoke and subcultured or not, compared to the TLR2 mRNA expression observed in the non-exposed cultures (Fig. 10). Interestingly, TLR2 expression levels in the smoke-exposed subcultured cells were comparable to those in the smoke-exposed non-subcultured cells. Similar results were obtained with HBD2 (Fig. 10). The level of HBD2 mRNA expression in the smoke-exposed subcultured cells was significantly (p<0.01) higher than that recorded by the non-exposed cells. However, no significant difference was recorded between the HBD mRNA expression levels in the smoke-exposed subcultured cells and those in the non-subcultured cells under the same conditions. This observation suggests that the stimulatory effect of cigarette smoke on HBD2 expression was maintained from one passage to another. Similar results were obtained with IL-6 mRNA expression (Fig. 10) levels, which were significant (p<0.01), as high levels of IL-6 mRNA were found in the smoke-exposed subcultured cells and smoke-exposed non-subcultured cells compared to the control cells. HBD2 and IL-6 mRNA modulations were confirmed by protein analyses (Fig. 11) showing high levels of HBD2 and IL-6 secretions by gingival epithelial cells exposed to cigarette smoke for 15 min then subcultured 3 different times.

## Discussion

The most common type of oral cancer is squamous cell carcinoma, which develops from the stratified squamous epithelium lining the mouth and pharynx [7], [8], [43]. This form of cancer accounts for approximately 90% of oral malignancies and may be promoted by cigarette smoke [43], [44]. Tobacco combustion products contain both gaseous and particulate components [45], [46]. Cigarette smoke constituents first interface with the immune system at the mucosal surface lining with the oral cavity as an initial contact leading to the possible deregulation of innate immunity in the oral mucosa. This hypothesis is supported by this study, which demonstrates an increase of TLR2, 4, and 6 expression by gingival epithelial cells following exposure to the gaseous and particulate components of cigarette constituents.

This up regulation of TLR expression by gingival epithelial cells exposed to whole cigarette smoke is reported here for the first time and supports previously reported data with various other cell types. Indeed, it was reported that cigarette smoke extracts increased TLR4 expression by the bronchial epithelial cell line 16-HBE [47], and cigarette smoke condensate was shown to promote the TLR4/MyD88 signaling-dependent production of inflammatory cytokines including IL-α and pro-IL-1β [48].

In our study, however, the increased TLR2 expression by whole cigarette smoke contradicts previously reported data [47]–[49]. This difference may be explained by the cell type used and the experimental conditions. TLR2 modulation in gingival epithelial cells by cigarette smoke may be explained by the capacity of these cells to sense different harmful agents, such as bacteria, or chemicals such as those found in cigarette smoke.

The immune response function of epithelial cells also involves TLR6 [50]. Following contact with various agents including cigarette smoke, TLR6 may be modulated. As the first study of this phenomenon, we demonstrated an increase in TLR6 expression by gingival epithelial cells following exposure to whole cigarette smoke, which may suggest that, similar to TLR2 and TLR4, TLR6 expression represents a defensive strategy used by epithelial cells to overcome the effect of cigarette smoke through a pro-inflammatory mediating response.

A similar activation of TLRs by cigarette smoke was reported with lymphocytes collected from patients suffering from chronic obstructive pulmonary disease (COPD) [51]. TLR activation was also reported in *H. pylori*-associated gastric cancer [52]. Furthermore, the high-mobility group box-1 (HMGB1) protein, a cytokine-like factor expressed by multiple mammalian cells, is known to be strongly up regulated in breast cancer, colon cancer, melanoma, pancreatic cancer, and prostate cancer, and this up regulation activates TLR2 and TLR4 expression in immune cells, thereby facilitating cancer progression and metastasis [53]. Overall, our study and others [53] in the literature point to a role of TLRs in cancer and suggest that this phenomenon may be stimulated by cigarette smoke. The activation of TLR may lead to the activation of multiple intracellular signals, including MAP kinases, as shown by ERK1/2, p38 and JNK phosphorylation, which was confirmed by the use of specific MAP Kinase and NFκB inhibitors. The involvement of TLRs through ERK1/2 and JNK was also reported with cell stimulation by either LPS or bacteria [54]. These MAP kinases appear to be the most solicited, as p38 MAP kinase was not involved. Non-activation of p38 was previously reported in an HBE cell line exposed to cigarette smoke extracts [47].

The implication of ERK1/2 and JNK following TLR modulation is critical to the initiation of the proinflammatory response through the production of such cationic peptides as human β-defensins [55], [56]. Exposure of gingival epithelial cells to whole cigarette smoke promoted HBD2 and HBD3 expression through the ERK1/2 MAP kinase and NFκB pathways. These data are not in agreement with previous reports [55], [56] showing that acrolein (a major component of cigarette smoke) and cigarette smoke extract suppressed HBD2 expression by sinonasal epithelial cells and gingival epithelial cells. Furthermore, using pharyngeal washing fluid and sputum from current and former smokers with acute pneumonia, Herr *et al.* ^57^ reported decreased HBD2 levels and demonstrated that smoke exposure reduced HBD2 expression *in vitro* in airway epithelium in response to bacterial stimulation [57]. In contrast, with a murine model, Shibata *et al.* ^58^ reported increased HBD2 mRNA expression in lung tissue after exposure to cigarette smoke for six months [58]. Similar results were reported with a rat model [59]. These conflicting findings may be explained by the different experimental model used in each study. Furthermore, the cell type may be of considerable importance, as gingival epithelial cells and bronchial epithelial cells may react differently when exposed to the same stimulus, including cigarette smoke.

In our study, whole cigarette smoke led to an increase in HBD3 expression and secretion by the gingival epithelial cells. This study is the first to report such a response. The increased HBD3 supports results obtained with HBD2 following cell exposure to whole cigarette smoke, thus confirming the proinflammatory response of gingival cells following exposure to cigarette smoke.

The smoke-induced expression of HBD2 and HBD3 both involved the ERK1/2 and NFκB signaling pathways. This result is consistent with previously reported studies in which cigarette smoke extracts activated the ERK pathway in human dendritic cells [60] and human gingival epithelial cells exposed to nicotine [61], and the NFκB pathway in human bronchial epithelial cells [62].

TLR and HBD modulated expression by whole cigarette smoke could be supported by pro-inflammatory cytokine (IL-1β and IL-6) induction. Indeed, cell exposure to whole cigarette smoke resulted in a significant increase in IL-1β expression and production. This increase also involved the ERK1/2 MAP kinase and NFκB pathways, which is consistent with previously reported data showing the active role of IL-1β in the pathogenesis of multiple pulmonary diseases, such as pulmonary infection, fibrosis, cancer, and emphysema [63], [64]. The implication of IL-1β was also confirmed in an animal model, showing that cigarette smoke-induced emphysema was reduced in mice that were null for the receptors of IL-1β and TNF-α [63]. Furthermore, cigarette smoke extract was shown to up regulate IL-1β expression by pulmonary epithelial cells [65]. IL-6 up regulation was also observed following cell exposure to cigarette smoke. Gingival epithelial cells exposed to whole cigarette smoke demonstrated a significant increase of IL-6 mRNA expression and protein secretion. As a pro-inflammatory cytokine, an increase of IL-6 was also reported in smokers [66], in HBE16 human airway epithelial cells [67], and in human bronchial epithelial cells [68].

The results of this study indicate that the inflammatory process launched by the gingival epithelial cells following exposure to whole cigarette smoke involves ERK1/2 and NFκB as key pathways, and to a lesser extent, the JNK MAP kinase in the case of the TLRs. The activity of several MAP kinase pathways was measured by Western blot and immunofluorescence analyses on the cigarette smoke-exposed cells, revealing a marked phosphorylation of ERK1/2 and a translocation of NFκB from the cytoplasm to the nucleus. These signaling pathways were also involved in TLR, HBD, and pro-inflammatory cytokine regulation in the smoke-exposed gingival epithelial cells, which suggests that these cells activated their innate immune response through the ERK1/2 and NFκB pathways and therefore play a crucial role in protecting against the effect of the cigarette smoke. These data are consistent with reports that cigarette smoke induces mucus hypersecretion through the ERK1/2 and JNK pathways [69], [70].

The effect of cigarette smoke on gingival epithelial cells may be either transient or maintained over a long period of time. For this reason, gingival epithelial cells were exposed to cigarette smoke and then maintained for long culture periods. Reported here for the first time, it is interesting to note that during ten days of culture, with three subculture times, the high levels of TLR-2, HBD2, and IL-6 expression were maintained up to 10 days post-exposure to cigarette smoke and that these levels were comparable to those recorded with smoke-exposed cells cultured for short periods of time (1, 3 and 6 h). Maintaining a high pro-inflammatory status for such a long time may be physiologically consequential, as cells are immunologically active through TLR2, HBD, and IL-6 expression [71]. This innate immunity status may be important for the early sensing and control of bacterial infections [72]. However, continuous TLR2 expression over a long period may be a sign of cancer, as reported in cancer tissue studies [73]. In conclusion, this study reveals new potential mechanisms by which cigarette smoke may contribute to the onset of the oral innate immunity reaction, as the smoke compounds increased the expression of specific innate immunity receptors. In turn, this expression promoted a pro-inflammatory response through the expression and production of cationic peptides and the pro-inflammatory cytokines IL-1β and IL-6. These mediators may be efficient at limiting microbial invasion; however, these events may also contribute to generating negative feedback by inducing gingival epithelial cell overstimulation and solicitation, which may ultimately lead to epithelial oral squamous cell carcinoma development.

## References

[1] Pauwels RA, Rabe KF (2004) Burden and clinical features of chronic obstructive pulmonary disease (COPD). Lancet 364: 613–620. 15313363 10.1016/S0140-6736(04)16855-4

[2] Proctor I, Sharma V, Khoshzaban M, Winstanley A (2012) Does smoking kill? A study of death certification and smoking. J Clin Pathol 65(2): 129–32. 22024242 10.1136/jclinpath-2011-200299

[3] Moshammer H, Hoek G, Luttmann-Gibson H, Neuberger MA, Antova T, et al (2006) Parental smoking and lung function in children: an international study. Am J Respir Crit Care Med 173: 1255–1263. 16484675 10.1164/rccm.200510-1552OC

[4] Hecht SS (2006) Cigarette smoking: cancer risks, carcinogens, and mechanisms. Langenbecks Arch Surg 391: 603–613. 17031696 10.1007/s00423-006-0111-z

[5] Parkin DM, Bray F, Ferlay J, Pisani P (2005) Global cancer statistics 2002. CA Cancer J Clin 55: 74–108. 15761078 10.3322/canjclin.55.2.74

[6] DeMarini MD (2004) Genotoxicity of tobacco smoke and tobacco smoke condensate: a review. Mutat Res 567: 447–74. 15572290 10.1016/j.mrrev.2004.02.001

[7] Taybos G (2003) Oral changes associated with tobacco use. Am J Med Sci 326: 179–82. 14557730 10.1097/00000441-200310000-00005

[8] Muscat JE, Ahn K, Richie JP Jr, Stellman SD (2011) Nicotine dependence phenotype, time to first cigarette, and risk of head and neck cancer. Cancer 117(23): 5377–82. 21826643 10.1002/cncr.26235 PMC3213279

[9] Nagler R, Ben-Izhak O, Savulescu D, Krayzler E, Akrish S, et al (2010) Oral cancer, cigarette smoke and mitochondrial 18kDa translocator protein (TSPO) - In vitro, in vivo, salivary analysis. Biochim Biophys Acta 1802(5): 454–61. 20085808 10.1016/j.bbadis.2010.01.008

[10] Starrett W, Blake DJ (2011) Sulforaphane inhibits de novo synthesis of IL-8 and MCP-1 in human epithelial cells generated by cigarette smoke extract. J Immunotoxicol 8(2): 150–8. 21401388 10.3109/1547691X.2011.558529

[11] Simone RE, Russo M, Catalano A, Monego G, Froehlich K, et al (2011) Lycopene inhibits NF-kB-mediated IL-8 expression and changes redox and PPARγ signalling in cigarette smoke-stimulated macrophages. PLoS One 6(5): e19652. 21625550 10.1371/journal.pone.0019652 PMC3098254

[12] Balansky R, D’Agostini F, Micale RT, La Maestra S, Steele VE, et al (2012) Dose-related cytogenetic damage in pulmonary alveolar macrophages from mice exposed to cigarette smoke early in life. Arch Toxicol 86(3): 509–16. 21989788 10.1007/s00204-011-0765-3

[13] Khorram O, Han G, Magee T (2010) Cigarette smoke inhibits endometrial epithelial cell proliferation through a nitric oxide-mediated pathway. Fertil Steril 93(1): 257–63. 19022425 10.1016/j.fertnstert.2008.09.074

[14] Brown GP, Iwamoto GK, Monick MM, Hunninghake GW (1989) Cigarette smoking decreases interleukin-1 release by human alveolar macrophages. Am J Physiol 256: C260–C264. 2784033 10.1152/ajpcell.1989.256.2.C260

[15] Lee J, Taneja V, Vassallo R (2012) Cigarette smoking and inflammation: cellular and molecular mechanisms. J Dent Res 91(2): 142–9. 21876032 10.1177/0022034511421200 PMC3261116

[16] Tsoumakidou M, Tsiligianni I, Tzanakis N (2011) Mechanisms of altered cell immunity, cytotoxicity in COPD. Curr Drug Targets 12(4): 450–9. 21194409 10.2174/138945011794751546

[17] Gorr SU (2012) Antimicrobial peptides in periodontal innate defense. Front Oral Biol 15: 84–98. 22142958 10.1159/000329673 PMC3704226

[18] Weinberg A, Krisanaprakornkit S, Dale BA (1998) Epithelial antimicrobial peptides: review and significance for oral applications. Crit Rev Oral Biol Med 9(4): 399–414. 9825219 10.1177/10454411980090040201

[19] Janardhanam SB, Prakasam S, Swaminathan VT, Kodumudi KN, Zunt SL, et al.. (2011) Differential expression of TLR-2 and TLR-4 in the epithelial cells in oral lichen planus. Arch Oral Biol Nov 24. 10.1016/j.archoralbio.2011.10.013 22119043

[20] Rouabhia M, Mukherjee PK, Lattif AA, Curt S, Chandra J, et al (2011) Disruption of sphingolipid biosynthetic gene IPT1 reduces Candida albicans adhesion and prevents activation of human gingival epithelial cell innate immune defense. Med Mycol 49(5): 458–66. 21091155 10.3109/13693786.2010.535031

[21] Singh RK, Srivastava A, Singh N (2012) Toll-like receptor signaling: A perspective to develop vaccine against leishmaniasis. Microbiol Res Feb 9. 10.1016/j.micres.2012.01.002 22326459

[22] Hajishengallis G (2011) Immune evasion strategies of Porphyromonas gingivalis. J Oral Biosci 53(3): 233–240. 22162663 10.2330/joralbiosci.53.233 PMC3231999

[23] Gillaux C, Méhats C, Vaiman D, Cabrol D, Breuiller-Fouché M (2011) Functional screening of TLRs in human amniotic epithelial cells. J Immunol 187(5): 2766–74. 21775685 10.4049/jimmunol.1100217

[24] Eckmann L (2006) Sensor molecules in intestinal innate immunity against bacterial infections. Curr Opin Gastroenterol 22: 95–101. 16462163 10.1097/01.mog.0000208458.38772.2a PMC2695762

[25] Vu AT, Baba T, Chen X, Le TA, Kinoshita H, et al (2010) Staphylococcus aureus membrane and diacylated lipopeptide induce thymic stromal lymphopoietin in keratinocytes through the Toll-like receptor 2-Toll-like receptor 6 pathway. J Allergy Clin Immunol 126(5): 985–93. 21050945 10.1016/j.jaci.2010.09.002

[26] Lim DM, Narasimhan S, Michaylira CZ, Wang ML (2009) TLR3-mediated NF-{kappa}B signaling in human esophageal epithelial cells. Am J Physiol Gastrointest Liver Physiol 297(6): G1172–80. 19779021 10.1152/ajpgi.00065.2009 PMC2850089

[27] Miller LS, Sørensen OE, Liu PT, Jalian HR, Eshtiaghpour D, et al (2005) TGF-alpha regulates TLR expression and function on epidermal keratinocytes. J Immunol 174(10): 6137–43. 15879109 10.4049/jimmunol.174.10.6137

[28] Gao N, Kumar A, Jyot J, Yu FS (2010) Flagellin-induced corneal antimicrobial peptide production and wound repair involve a novel NF-kappaB-independent and EGFR-dependent pathway. PLoS One 5(2): e9351. 20195469 10.1371/journal.pone.0009351 PMC2829077

[29] Lebre MC, van der Aar AM, van Baarsen L, van Capel TM, Schuitemaker JH, et al (2007) Human keratinocytes express functional Toll-like receptor 3, 4, 5, and 9. J Invest Dermatol 127(2): 331–41. 17068485 10.1038/sj.jid.5700530

[30] Décanis N, Savignac K, Rouabhia M (2009) Farnesol promotes epithelial cell defense against Candida albicans through Toll-like receptor 2 expression, interleukin-6 and human beta-defensin 2 production. Cytokine 45(2): 132–40. 19121950 10.1016/j.cyto.2008.11.011

[31] Correa-Costa M, Braga TT, Semedo P, Hayashida CY, Bechara LR, et al (2011) Pivotal role of Toll-like receptors 2 and 4, its adaptor molecule MyD88, and inflammasome complex in experimental tubule-interstitial nephritis. PLoS One 6(12): e29004. 22194975 10.1371/journal.pone.0029004 PMC3237574

[32] Brown J, Wang H, Hajishengallis GN, Martin M (2011) TLR-signaling networks: an integration of adaptor molecules, kinases, and cross-talk. J Dent Res 90(4): 417–27. 20940366 10.1177/0022034510381264 PMC3075579

[33] Kawai T, Akira S (2007) TLR signaling. Semin Immunol 19: 24–32. 17275323 10.1016/j.smim.2006.12.004

[34] Raman M, Chen W, Cobb MH (2007) Differential regulation and properties of MAPKs. Oncogene 26: 3100–3112. 17496909 10.1038/sj.onc.1210392

[35] Lee MS, Kim YJ (2007) Signaling pathways downstream of pattern recognition receptors and their cross talk. Annu Rev Biochem 76: 447–480. 17328678 10.1146/annurev.biochem.76.060605.122847

[36] Malemud CJ (2007) Inhibitors of stress-activated protein/mitogen activated protein kinase pathways. Curr Opin Pharmacol 7: 339–343. 17398158 10.1016/j.coph.2006.11.012

[37] Maunders H, Patwardhan S, Phillips J, Clack A, Richter A (2007) Human bronchial epithelial cell transcriptome: gene expression changes following acute exposure to whole cigarette smoke in vitro. Am J Physiol Lung Cell Mol Physiol 292(5): L1248–56. 17220372 10.1152/ajplung.00290.2006

[38] Pelaia G, Cuda G, Vatrella A, Gallelli L, Fratto D, et al (2004) Effects of hydrogen peroxide on MAPK activation, IL-8 production and cell viability in primary cultures of human bronchial epithelial cells. J Cell Biochem 93(1): 142–52. 15352171 10.1002/jcb.20124

[39] Palozza P, Serini S, Di Nicuolo F, Boninsegna A, Torsello A, et al (2004) beta-Carotene exacerbates DNA oxidative damage and modifies p53-related pathways of cell proliferation and apoptosis in cultured cells exposed to tobacco smoke condensate. Carcinogenesis 25(8): 1315–25. 15073048 10.1093/carcin/bgh142

[40] Semlali A, Chakir J, Goulet JP, Chmielewski W, Rouabhia M (2011) Whole cigarette smoke promotes human gingival epithelial cell apoptosis and inhibits cell repair processes. J Periodontal Res 46(5): 533–41. 21517857 10.1111/j.1600-0765.2011.01370.x

[41] Semlali A, Leung KP, Curt S, Rouabhia M (2011) Antimicrobial decapeptide KSL-W attenuates Candida albicans virulence by modulating its effects on Toll-like receptor, human β-defensin, and cytokine expression by engineered human oral mucosa. Peptides 32(5): 859–67. 21291939 10.1016/j.peptides.2011.01.020

[42] Imani Fooladi AA, Mousavi SF, Seghatoleslami S, Yazdani S, Nourani MR (2011) Toll-like receptors: role of inflammation and commensal bacteria. Inflamm Allergy Drug Targets 10(3): 198–207. 21428911 10.2174/187152811795564064

[43] Proia NK, Paszkiewicz GM, Nasca MA, Franke GE, Pauly JL (2006) Smoking and smokeless tobacco-associated human buccal cell mutations and their association with oral cancer–a review. Cancer Epidemiol Biomarkers Prev 15(6): 1061–77. 16775162 10.1158/1055-9965.EPI-05-0983

[44] Hecht SS (2003) Tobacco carcinogens, their biomarkers and tobacco-induced cancers. Nat Rev Cancer 3: 733–44. 14570033 10.1038/nrc1190

[45] Bluhm AL, Weinstein J, Sousa JA (1971) Free radicals in tobacco smoke. Nature 229(5285): 500. 10.1038/229500a0 4925214

[46] Witschi H (2005) Carcinogenic activity of cigarette smoke gas phase and its modulation by beta-carotene and N-acetylcysteine. Toxicol Sci 84(1): 81–7. 15564316 10.1093/toxsci/kfi043

[47] Pace E, Ferraro M, Siena L, Melis M, Montalbano AM, et al (2008) Cigarette smoke increases Toll-like receptor 4 and modifies lipopolysaccharide-mediated responses in airway epithelial cells. Immunology 124(3): 401–11. 18217953 10.1111/j.1365-2567.2007.02788.x PMC2440834

[48] Doz E, Noulin N, Boichot E, Guénon I, Fick L, et al (2008) Cigarette smoke-induced pulmonary inflammation is TLR4/MyD88 and IL-1R1/MyD88 signaling dependent. J Immunol 180(2): 1169–78. 18178857 10.4049/jimmunol.180.2.1169

[49] Droemann D, Goldmann T, Tiedje T, Zabel P, Dalhoff K, et al (2005) Toll-like receptor 2 expression is decreased on alveolar macrophages in cigarette smokers and COPD patients. Respir Res 8 6: 68. 10.1186/1465-9921-6-68 PMC1187924 16004610

[50] Depaolo RW, Tang F, Kim I, Han M, Levin N, et al (2008) Toll-like receptor 6 drives differentiation of tolerogenic dendritic cells and contributes to LcrV-mediated plague pathogenesis. Cell Host Microbe 16 4(4): 350–61. 10.1016/j.chom.2008.09.004 PMC2633104 18854239

[51] Nadigel J, Préfontaine D, Baglole CJ, Maltais F, Bourbeau J, et al (2011) Cigarette smoke increases TLR4 and TLR9 expression and induces cytokine production from CD8^+^ T cells in chronic obstructive pulmonary disease. Respir Res 9 12: 149. 10.1186/1465-9921-12-149 PMC3260101 22070100

[52] Jüttner S, Cramer T, Wessler S, Walduck A, Gao F, et al (2003) *Helicobacter pylori* stimulates host cyclooxygenase-2 gene transcription: critical importance of MEK/ERK-dependent activation of USF1/−2 and CREB transcription factors. Cell Microbiol 5: 821–834. 14531897 10.1046/j.1462-5822.2003.00324.x

[53] Poser I, Bosserhoff AK (2004) Transcription factors involved in development and progression of malignant melanoma. Histol Histopathol 19: 173–88. 14702186 10.14670/HH-19.173

[54] De Nardo D, De Nardo CM, Nguyen T, Hamilton JA, Scholz GM (2009) Signaling crosstalk during sequential TLR4 and TLR9 activation amplifies the inflammatory response of mouse macrophages. J Immunol 183(12): 8110–8. 19923461 10.4049/jimmunol.0901031

[55] Lee WK, Ramanathan M Jr, Spannhake EW, Lane AP (2007) The cigarette smoke component acrolein inhibits expression of the innate immune components IL-8 and human b-defensin 2 by sinonasal epithelial cells. Am J Rhinol 21: 658–663. 18201443 10.2500/ajr.2007.21.3094

[56] Mahanonda R, Sa-Ard-Iam N, Eksomtramate M, Rerkyen P, Phairat B, et al (2009) Cigarette smoke extract modulates human b-defensin-2 and interleukin-8 expression in human gingival epithelial cells. J Periodontal Res 44: 557–564. 19438974 10.1111/j.1600-0765.2008.01153.x

[57] Herr C, Beisswenger C, Hess C, Kandler K, Suttorp N, et al (2009) Suppression of pulmonary innate host defence in smokers. Thorax 64: 144–149. 18852155 10.1136/thx.2008.102681

[58] Shibata Y, Abe S, Inoue S, Takabatake N, Igarashi A, et al (2008) Altered expression of antimicrobial molecules in cigarette smoke-exposed emphysematous mice lungs. Respirology 13: 1061–1065. 18699806 10.1111/j.1440-1843.2008.01362.x

[59] Chen L, Sun BB, Wang T, Wang X, Li JQ, et al (2010) Cigarette smoke enhances {beta}-defensin 2 expression in rat airways via nuclear factor-{kappa}B activation. Eur Respir J. 36(3): 638–45. 10.1183/09031936.00029409 20150208

[60] Kroening PR, Barnes TW, Pease L, Limper A, Kita H, et al (2008) Cigarette smoke-induced oxidative stress suppresses generation of dendritic cell IL-12 and IL-23 through ERK-dependent pathways. J Immunol 181(2): 1536–47. 18606709 10.4049/jimmunol.181.2.1536 PMC2819390

[61] Kashiwagi Y, Yanagita M, Kojima Y, Shimabukuro Y, Murakami S (2011) Nicotine up-regulates IL-8 expression in human gingival epithelial cells following stimulation with IL-1β or *P. gingivalis* lipopolysaccharide via nicotinic acetylcholine receptor signalling. Arch Oral Biol Nov 24. 10.1016/j.archoralbio.2011.10.007 22119045

[62] Profita M, Bonanno A, Montalbano AM, Ferraro M, Siena L (2011) Cigarette smoke extract activates human bronchial epithelial cells affecting non-neuronal cholinergic system signalling in vitro. Life Sci 89(1–2): 36–43. 21620875 10.1016/j.lfs.2011.04.025

[63] Hogg JC, Chu F, Utokaparch S, Woods R, Elliott WM, et al (2004) The nature of small-airway obstruction in chronic obstructive pulmonary disease. N Engl J Med 350: 2645–2653. 15215480 10.1056/NEJMoa032158

[64] Barnes PJ, Shapiro SD, Pauwels RA (2003) Chronic obstructive pulmonary disease: molecular and cellular mechanisms. Eur Respir J 22: 672–688. 14582923 10.1183/09031936.03.00040703

[65] Reynolds PR, Cosio MG, Hoidal JR (2006) Cigarette smoke-induced Egr-1 upregulates proinflammatory cytokines in pulmonary epithelial cells. Am J Respir Cell Mol Biol 35(3): 314–9. 16601242 10.1165/rcmb.2005-0428OC PMC2643284

[66] Herfs M, Hubert P, Poirrier AL, Vandevenne P, Renoux V, et al.. (2012) Pro-inflammatory Cytokines Induce Bronchial Hyperplasia and Squamous Metaplasia in Smokers: Implications for COPD Therapy. Am J Respir Cell Mol Biol Feb 16. 10.1165/rcmb.2011-0353OC 22343222

[67] Li Q, Zhou X, Kolosov VP, Perelman JM (2010) Nicotine suppresses inflammatory factors in HBE16 airway epithelial cells after exposure to cigarette smoke extract and lipopolysaccharide. Transl Res 156(6): 326–34. 21078494 10.1016/j.trsl.2010.09.001

[68] Liu X (2007) STAT3 activation inhibits human bronchial epithelial cell apoptosis in response to cigarette smoke exposure. Biochem Biophys Res Commun 353(1): 121–6. 17173857 10.1016/j.bbrc.2006.11.147

[69] Yu H, Li Q, Kolosov VP, Perelman JM, Zhou X (2011) Regulation of cigarette smoke-induced mucin expression by neuregulin1β/ErbB3 signalling in human airway epithelial cells. Basic Clin Pharmacol Toxicol 109(1): 63–72. 21332945 10.1111/j.1742-7843.2011.00686.x

[70] Lee SY, Kang EJ, Hur GY, Jung KH, Jung HC, et al (2006) Peroxisome proliferator-activated receptor-gamma inhibits cigarette smoke solution-induced mucin production in human airway epithelial (NCI-H292) cells. Am J Physiol Lung Cell Mol Physiol 291(1): L84–90. 16443643 10.1152/ajplung.00388.2005

[71] Kumar A, Zhang J, Yu FS (2006) Toll-like receptor 2-mediated expression of beta-defensin-2 in human corneal epithelial cells. Microbes Infect 8(2): 380–9. 16242370 10.1016/j.micinf.2005.07.006 PMC2666383

[72] Santaolalla R, Abreu MT (2012) Innate immunity in the small intestine. Curr Opin Gastroenterol 28(2): 124–9. 22241076 10.1097/MOG.0b013e3283506559 PMC3502878

[73] Nihon-Yanagi Y, Terai K, Murano T, Matsumoto T, Okazumi S (2012) Tissue expression of Toll-like receptors 2 and 4 in sporadic human colorectal cancer. Cancer Immunol Immunother 61(1): 71–7. 21845432 10.1007/s00262-011-1085-4 PMC3249192

